# Is open decortication superior to fibrinolytic therapy as a first line treatment in the management of pleural empyema?

**DOI:** 10.12669/pjms.322.9676

**Published:** 2016

**Authors:** Sultan Ahmed, Hammad Azam, Imran Basheer

**Affiliations:** 1 Sultan Ahmed (FCPS General Surgery), Assistant Professor of General Surgery, Sheikh Zayed Medical College & Hospital, Rahim Yaar Khan, Pakistan; 2 Hammad Azam (FCPS General Surgery), Assistant Professor of Cardiac Surgery, Sheikh Zayed Medical College & Hospital, Rahim Yaar Khan, Pakistan; 3 Imran Basheer (FCPS Pulmonology), Assistant Professor of Pulmonology, Sheikh Zayed Medical College & Hospital, Rahim Yaar Khan, Pakistan

**Keywords:** Empyema, Open Decortication, Fibrinolytic therapy

## Abstract

**Objective:**

To confirm that either Fibrinolytic therapy or open decortication which of the two is an effective First line treatment of pleural empyema.

**Methods:**

This prospective comparative study was conducted in the department of surgery Sheikh Zayed Medical College and Hospital, Rahim Yaar Khan. Seventy eight (78) patients were included in this study. There were two groups of patients; Group I (n=35) patients treated with fibrinolytic therapy, Group II (n=43) patients treated with open decortication. Data was entered and analyzed in SPSS v16. Student’s t-test was used for comparison of quantitative variables. Chi-square and Fisher’s Exact test were used for comparison of qualitative variables. P-value ≤ 0.05 was taken as significant difference.

**Results:**

There was no significant difference in base baseline characteristics of patients of Group I and II. Incidence of comorbidities was also same between the groups. Most of the patients in Group I and II were in empyema stage III. Fluid cultures was positive in 33 (94.3%) patients in group I and 39 (90.7%) patients in group II. 30 (85.7%) was successfully treated using fibrinolytic therapy but this therapy failed in five (14.3%) patients, two of these patients expired within the hospital. There was only one (2.3%) treatment failure in open decortication Group that patient expired within the hospital (p-value 0.04). Overall duration of hospitalization was significantly high in fibrinolytic group, this was 17.6± 1.95 days versus 12.09± 2.18 days in open decortication group (p-value<0.0001). There was no significant difference regarding operative mortality within the two groups.

**Conclusion:**

Open Drainage is associated with better outcomes as compared to fibrinolytic therapy when used as a First line treatment of empyema.

## INTRODUCTION

The presence of purulent fluid within the pleural space is known as pleural empyema,^1^ caused by invasions of pathogens into the pleural space. The incidence of pleural empyema has been increasing worldwide in all age groups^2^ which has significantly increased the mortality and morbidity associated with pleural infections. There are three stages of empyema described by The American Thoracic Society; Stage I (exudative stage), Stage II (fibrinopurulent stage), Stage III (organizing stage).^3^ Treatment of empyema ranges from simple antibiotic administration to insertion of pigtail catheters, tube thoracostomy or surgical intervention e.g. open drainage and Video-assisted thoracoscopic surgery.^4^ Stage I can be easily treated using closed chest drainage and optimal antibiotic therapy with a success rate of about 80%.^5^ But in stage II and III antibiotics alone are not much effective. Surgical treatment is an alternative strategy for these patients. The two commonly used modalities are thoracostomy and open decortication. Multiple studies have shown that fibrinolytic therapy along with tube thoracostomyis an effective treatment for the management of fibrinopulurent and organizing empyema with a success rate ranging from 38-100%.^6^ Fibrinolytic therapy has shown to be superior to chest tube drainage only.^7^,^8^ When these therapies fails the ultimate treatment is surgical intervention.

The First line treatment of empyema thoracis still remains controversial. The present study aimed to evaluate either fibrinolytic therapy or open decortication which of these two should be the treatment of choice for the management of fibrinopurulent or organizing empyema.

## METHODS

After approval from the ethical committee of the hospital, this prospective comparative study was conducted in the department of general surgery Sheikh Zayed Medical College and Hospital, Rahim Yaar Khan. An informed consent was taken from the patient itself or the legal guardian of the patient. The patients were divided into two groups using draw randomization technique. A total number of 78 patients were included in this study. There were two groups of patients; Group I (n=35) patients treated with fibrinolytic therapy, Group II (n=43) patients treated with open decortication.

### Surgical Technique

In fibrinolytic group a 14 Fr chest tube was inserted using Seldinger technique. Tubes were placed using local anesthesia with sedation. Streptokinase (SK) was used for fibrinolysis. 250,000 U of SK was mixed with 100 ml of normal saline. The fluid was directly injected into the tube with a dwell time of about four hours. After that the tube was manually aspirated and then released into an underwater seal for passive drainage. The Streptokinase doses were repeated after every 24 hours up to a maximum period of 14 days. The total doses of SK administrated was determined by the patient response. The chest drains were removed when the daily drainage falls below 50 ml or the chest radiograph showed resolution of the pleural collection. If there was still drainage after 14 days of fibrinolytic therapy, the open surgical drainage was done.

In open decortication group, postero-lateral thoracotomy was performed by sparing serratus anterior muscles. Fibrin septae were cut, fissures ware dissected free and maximum evacuation of purulent fluid was done. Complete decortication of visceral and parietal pleura was carried out to obtain maximum lung expansion in all patients. Two large bore 32 Fr chest tubes were inserted at the end of the procedure through separate incisions. Chest tubes were removed when there was no air leakage and when the drainage falls to less than 50 ml per day.

Data was entered and analyzed in SPSS v16. Mean and standard deviation was used to express quantitative variables. Number and percentage was used for qualitative variables. Student’s t-test was used for comparison of quantitative variables. Chi-square and Fisher’s Exact test were used for comparison of qualitative variables. P-value ≤0.05 was taken as significant difference.

## RESULTS

There was no significant difference in the base baseline characteristics of patients of Group I and II. Incidence of Comorbidities was also same between the groups. The most common comorbid pathologies were; smoking, diabetes and Ischemic Heart Disease (IHD). Most of the patients in Group I and II were in empyema stage III. There were 19 (54.3%) patients in Empyema stage III in Group I and 24 (55.8%) patients in Group II but this difference was statistically insignificant. Patients who presented to us mostly were of having Right sided empyema, we divided them equally into two groups to minimize confounding effect. There were 23 (65.7%) patients having right sided pleural empyema in group I and 28 (65.1%) in group II, only one patient was of bilateral pleural empyema and it was adjusted in open decortication group. Fluid cultures was positive in 33 (94.3%) patients in group I and 39 (90.7%) patients in group II and this difference was statistically insignificant (Table-I).

**Table-I:** Comparison of Baseline Characteristics.

Variable		Fibrinolytic therapy (Group I)	Open Decortication (Group II)	p-value
Age (Years) mean± SD		56.42± 8.21	55.53± 8.82	0.89
Gender n (%)	Male	29 (82.9)	38 (88.4)	0.49
	Female	06 (17.1)	5 (11.6)	
BMI Kg/m^2^ (mean± SD)		24.8± 3.9	25.8± 4.56	0.29
Comorbidities				
Smoking n (%)		12 (34.30	16 (37.2)	0.79
Diabetes n (%)		9 (25.7)	14 (32.6)	0.51
IHD n (%)		3 (8.6)	6 (14.0)	0.45
Empyema Stage n (%)	II	16 (45.7)	19 (44.2)	0.89
	III	19 (54.3)	24 (55.8)	
Empyema Location n (%)	Right	23 (65.7)	28 (65.1)	0.66
	Left	12 (34.3)	14 (32.6)	
	Bilateral	0 (0.0)	1 (1.3)	
Positive Pleural Fluid Culture n (%)		33 (94.3)	39 (90.7)	0.69

BMI= Body Mass Index, IHD=Ischemic Heart Disease, n=Number of patients.

Duration of symptoms of empyema before treatment was same between two groups. 30 (85.7%) was successfully treated using fibrinolytic therapy but this therapy failed in five (14.3%) patients, these patients underwent open decortication later on and out of these five two patients (5.7%) were expired within the hospital. Due to primary treatment failure these patients remained in the hospital for a longer duration of time. There was only one (2.3%) treatment failure in open decortication Group, these patients was of having bilateral pleural empyema and he expired within the hospital after undergoing 2^nd^ attempt of open decortication. There was no significant difference regarding operative mortality within the two groups. Overall duration of hospitalization was significantly high in fibrinolytic group, this was 17.6± 1.95 days versus 12.09± 2.18 days in open decortication group with highly significant p-value of <0.0001 (Table-II).

**Table-II:** Comparison of Outcome Variables.

Variable	Fibrinolytic therapy (Group I)	Open Decortication (Group II)	p-value
Duration of Symptoms to treatment d(mean± SD)	12.91± 1.01	13.95± 1.02	0.66
Treatment Success n (%)	30 (85.7)	42 (97.7)	0.04
Duration of Hospitalization d(mean± SD)	17.6± 1.95	12.09± 2.18	<0.0001
Survival n (%)	33 (94.3)	42 (97.7)	0.43

d=Days, n=Number of patients.

## DISCUSSION

Instillation of fibrinolytic agents into the pleural space offers the benefit of lysing the fibrin adhesion thereby promoting pleural fluid drainage and thus avoiding surgery. British Thoracic society and ACCP has recommended fibrinolytic therapy as management options for the treatment of empyema.^9^-^11^ Streptokinase and urokinase has been shown to be equally effective ass fibrinolytic agents.^12^ In this study we used streptokinase for fibrinolytic therapy.

Peter et al. has recommended fibrinolytic therapy as a First line treatment for the management of empyema in children.^13^ Some other researchers have also recommended that open drainage should be considered after failure of simple drainage.^14^,^15^ The aim of the present study was to find out that which therapy is most beneficial if used as a First line treatment in advanced stages of empyema. We found that there was a higher rate of treatment failure in patients who received fibrinolytic therapy as a First line of treatment and five patients (14.5%) was shifted to open decortication in this group after treatment failure, out of these two patients were expired. Wozniak et al. also demonstrated that failure of the First procedure is a strongest predictor of hospital death in the management of empyema.^4^ Our study supports the results of this study. A meta-analysis that followed ACCP guidelines have concluded that surgical treatment (VATS or open decortication) is superior when compared with thoracostomy and fibrinolytic therapy.^9^ Many studies have concluded that surgical decortication as a First line treatment or after a short trial of simple drainage results in excellent success rates and reduction in mortality in patients with advanced stages of empyema.^16^-^19^

Open drainage allow complete evacuation of pleural fluid by debridement and disruption of all loculations which is necessary for full lung expansion and resolution of infection.^19^ But it exposes patient to the effects of anesthesia and operation which can otherwise be avoided. But it is associated with higher rate of treatment success and less mortality. Our study support the results of many studies that have demonstrated higher success rates when surgical drainage was used as a 1^st^ line treatment.^20^,^21^ In this study we found that patients who underwent open decortication as a First line treatment for the management of empyema had a significantly shorter hospital stay, rate of treatment success and survival. Many other studies have concluded that surgical decortication is associated with shorter hospital stay.^18^,^22^,^23^ Our study have supported the results of these studies.

## CONCLUSION

Open Drainage is associated with better outcomes as compared to fibrinolytic therapy when used as a First line treatment of empyema.
